# Effects of let-7a microRNA and C–C chemokine receptor type 7 expression on cellular function and prognosis in esophageal squamous cell carcinoma

**DOI:** 10.1186/s12885-022-10178-2

**Published:** 2022-10-15

**Authors:** Masahiro Yura, Kazumasa Fukuda, Satoru Matsuda, Tomoyuki Irino, Rieko Nakamura, Hirofumi Kawakubo, Hiroya Takeuchi, Yuko Kitagawa

**Affiliations:** 1 Department of Surgery, Keio University School of Medicine, 35 Shinanomachi, Shinjuku-ku, Tokyo, 160-8582 Japan; 2 Department of Surgery, Hamamatsu University School of Medicine, 1-20-1 Handayama, Higashi-ku, Hamamatsu-shi, Shizuoka, 431-3192 Japan

**Keywords:** Esophageal squamous cell carcinoma, C–C chemokine receptor type 7, Let-7a microRNA, Invasive ability, Metastasis

## Abstract

**Background:**

C–C chemokine receptor type 7 (CCR7) participates in chemotactic and metastatic responses in various cancers, including in esophageal squamous cell carcinoma (ESCC). The microRNA (miRNA) let-7a suppresses migration and invasion of various types of cancer cells by downregulating *CCR7* expression.

**Methods:**

The expression levels of *CCR7* and let-7a were measured in the cell lines, tumor, and peritumoral tissues of ESCC patients. KYSE cell lines were transfected with synthetic let-7a miRNA and a let-7a miRNA inhibitor, and their *CCR7* expression levels as well as invasive ability were evaluated. A highly invasive cell line was established via an invasion assay, and *CCR7* expression level along with let-7a level was subsequently evaluated. Cancer cells overexpressing *CCR7* were injected subcutaneously into mice, and the animals were monitored for tumor growth along with lymph node metastasis.

**Results:**

A negative correlation between *CCR7* and let-7a expression was observed in the ESCC cell lines as well as in tissue samples from patients. Synthetic let-7a decreased *CCR7* expression level, while the let-7a inhibitor increased it. In vitro, the established highly invasive cancer cells with high and low levels of *CCR7* and let-7a expression, respectively, exhibited a greater invasive ability than the wild-type cell line. The cells were associated with tumor growth and lymph node metastasis in mice. Patients in the high-*CCR7*/low-let-7a group had the worst prognosis, with a five-year recurrence free survival (5-RFS) rate of 37.5%, followed by the high-*CCR7*/high-let-7a (5-RFS: 60.0%) and low-*CCR7* (5-RFS: 85.7%; *p* = 0.038) groups.

**Conclusions:**

The expression of *CCR7* was downregulated by let-7a miRNA in esophageal cancer cells. The decrease in let-7a expression level led to the increased expression level of *CCR7* in ESCC cells, consequently increasing their invasive ability and malignancy and resulting in a worse prognosis for ESCC patients.

Trial registration.

Retrospectively registered.

**Supplementary Information:**

The online version contains supplementary material available at 10.1186/s12885-022-10178-2.

## Background

Cancer incidence and mortality are rapidly increasing worldwide. In 2018, esophageal cancer ranked seventh and sixth in terms of incidence and overall mortality, respectively [1]. Although the survival rate of esophageal cancer has improved due to the development of multidisciplinary treatment, the five-year post-esophagectomy survival rate is approximately 50% [2, 3]. Therefore, there is an urgent need to investigate the mechanisms of cancer progression and metastasis to improve the prognosis of patients with esophageal cancer.

Chemokines regulate tumor cell proliferation, infiltration, and metastasis [4, 5]. In a previous study, we demonstrated the significant clinicopathological relationship and functional causality between C–C chemokine receptor type 7 (CCR7) expression and lymph node metastasis in patients with esophageal squamous cell carcinoma (ESCC) [6].

MicroRNAs (miRNAs) are non-coding small RNA molecules that can control the translation of mRNAs and regulate several cancer-related genes [7–9]. The miRNA let-7a suppresses various types of cancers [10–13]. For example, in breast cancer, let-7a suppresses the expression of CCR7 and reduces the ability of cancer cells to migrate and invade. Furthermore, a recent prospective study has reported that high let-7a expression level can be a predictive factor for favorable response to chemotherapy [14]. For gastric cancer, a lack of let-7a expression increases CCR7 expression level and is associated with metastasis, contributing to a poor prognosis [12]. Similarly, in esophageal cancer, plasma levels of let-7a miRNA are significantly lower in cancer patients than in healthy participants [15].

However, the effect of let-7a on molecular expression is still unclear, and it has not been confirmed whether the downregulation of let-7a miRNA expression is responsible for increased *CCR7* expression levels in ESCC tissues. Here, we investigated the relationship between *CCR7* and let-7a miRNA expression as well as the underlying regulatory mechanism, in esophageal cancer cell lines, tumor tissues, and peritumor tissues of patients with ESCC.

## Methods

### Tissue samples

Tissue samples were obtained during a biopsy from 17 ESCC patients at the Keio University School of Medicine in Japan. Immediately after the procedure, the samples were frozen in liquid nitrogen and stored at -70 °C until use.

### Esophageal cancer cell lines

For this study, we used six established ESCC cell lines from the KYSE series (KYSE-350, 510, 590, 1260, 1440, and 2400), purchased from the Japanese Collection of Research Bioresources Cell Bank of the National Institutes of Biomedical Innovation, Health, and Nutrition.

### Cell culture

Cells were maintained and cultured in a Roswell Park Memorial Institute 1640 medium supplemented with 10% fetal bovine serum, 100 units/mL penicillin, and 100 µg/mL streptomycin at 37 °C and 5% CO_2_ atmospheric content. For the experiments, cells were collected and prepared as single-cell suspensions in phosphate-buffered saline.

### Transfection

The sequence of double-stranded RNA (dsRNA) used in transfection experiments as scrambled small interfering RNA was 5′-UCACAACCUCCUAGAAAGAGUAGA-3′, that of synthetic let-7a-5p miRNA was 5′-UGAGGUAGUAGGUUGUAUAGUU-3′, while inhibitor let-7a-5p miRNA was 5′-UGAGGUAGUAGGUUGUAUAGUU-3′. These RNAs were synthesized by Applied Biosystems (Tokyo, Japan). The cell lines were transfected with dsRNA using the reagents Lipofectamine RNAiMAX and Lipofectamine 2000 (Invitrogen, Tokyo, Japan), according to the reagent manufacturer’s instructions. The cell lines were harvested 2 d after transfection and subjected to various analyses.

### Isolation and quantitative determination of expression level of let-7a

Isolation of let-7a miRNA from the cell lines was conducted using the mirVana™ miRNA Isolation Kit. The RNA concentration was quantified via the NanoDrop ND-1000 Spectrophotometer (Thermo Fisher Scientific, Tokyo, Japan). The complementary DNA (cDNA) was synthesized by the reverse transcription of let-7a miRNA. A quantitative reverse transcription polymerase chain reaction (RT-PCR) was conducted using ViiA™ 7 (Applied Biosystems); the thermal cycling consisted of an initial cycle of 2 min at 50 °C and 10 min at 95 °C, followed by 40 cycles for 15 s at 95 °C and 60 s at 60 °C. Relative let-7a expression level was quantified using the 2^−ΔΔCt^ method.

### Isolation of RNA and synthesis of single-stranded cDNA

Isolation of total RNA from cell lines was conducted using an RNeasy® Micro Kit (QIAGEN, Tokyo, Japan). The RNA concentration was quantified using a NanoDrop ND-1000 Spectrophotometer (Thermo Fisher Scientific). Then, synthesis of cDNA from total RNA was performed using an RNA-to-cDNA kit (Applied Biosystems). The quality and quantity of the cDNA samples were evaluated via standard electrophoresis and a NanoDrop ND-1000 Spectrophotometer (Thermo Fisher Scientific).

### Real-time PCR

The quantitative RT-PCR analysis was performed via ViiA™ 7 (Applied Biosystems) and the Fast SYBR® Green Master Mix (Applied Biosystems). Glyceraldehyde-3-phosphate dehydrogenase (*GAPDH*) and *CCR7* expression levels were evaluated using PCR primers. The thermal cycling consisted of an initial 20 s at 95 °C, followed by 40 cycles for 1 s at 95 °C and 20 s at 60 °C, using *GAPDH* as an internal control. The relative amount of *CCR7* expression in KYSE cell lines was calculated using the 2^−ΔΔCt^ method, wherein the KYSE-350 expression level was defined as 1.

### Let-7a transfection

A synthetic let-7a and a let-7a inhibitor were used for transfection. We diluted 15 μL of the Lipofectamine® RNAiMAX reagent (Life Technologies) in 2 mL of Opti-MEM® (Life Technologies), then added 2 mL of diluted let-7a miRNA (300 pmol) to diluted Lipofectamine® RNAiMAX and incubated the mixture for 5 min at room temperature. Next, we plated the transfection reagent onto a 10 cm plate, which was incubated for 10–20 min in a CO_2_ incubator at 37 °C. A suspension of 5 × 10^5^ cells in an antibiotic-free medium was added to the plate and incubated for 3 days in a CO_2_ incubator at 37 °C.

### Invasion assay

To create highly invasive cell lines, we used Corning BioCoat Matrigel Invasion Chambers containing a polyester membrane with 8 µm pores and a thin layer of a Matrigel Basement Membrane Matrix. We prepared a suspension of 2 × 10^4^ cells/mL in culture medium for 24-well chambers and incubated it in an invasion chamber for 72–96 h in a humidified tissue culture incubator at 37 °C in 5% CO_2_ atmosphere. After incubation, the non-invading cells were removed from the upper surface of the membrane while the cells in the lower surface were collected and reseeded to the chamber. After repeating this process six times, a highly invasive cell population was obtained and isolated using a cell dissociation solution, solubilized, and stained using a staining solution. The number of infiltrated cells were quantified by measuring the absorbance of the solution.

### Development of lymph node metastasis model

A lymph node metastasis model was developed through the following steps: 1) a xenograft tumor was created subcutaneously by injecting 1 × 10^7^ wild-type and invasive KYSE-510 cells into five-week-old mice, visualized with Green Fluorescent Protein (GFP) transfection; 2) after growth (4–6 weeks post injection), the resulting tumors were resected and cut into small pieces (approximately 2 to 8 mm^3^), then transplanted into the elbows of different five-week-old mice; 3) the accessory axillary lymph nodes were removed and examined 8 weeks after transplantation.

### Statistical analysis

Statistical analysis was done using the IBM SPSS statistics 26.0 software. The continuous variables were expressed as mean ± SD and compared with the Student’s *t*-test. Recurrence-free survival (RFS) was measured from the time of surgery until the recurrence of tumor or death, whichever came first. The RFS curves were estimated with the Kaplan–Meier method and compared by the log-rank test.

Univariate analysis was then performed using Cox’s proportional hazard model, and a p-value of 0.05 was considered as statistically significant.

## Results

### Expression of *CCR7* and let-7a in esophageal cancer cell lines

**Fig. 1:**
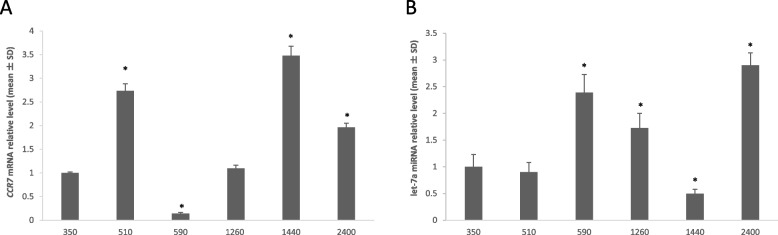
Quantitative RT-PCR analysis. **A** *CCR7* expression in KYSE cell lines. **B** Let-7a expression in KYSE cell lines. (* *p* < 0.05 versus KYSE 350 cells)

Using quantitative RT-PCR analysis, *CCR7* mRNA expression was confirmed in all esophageal cancer cell lines (Fig. 1A), and the level of let-7a miRNA expression was also determined (Fig. 1B). KYSE-510 and 1440 exhibited significantly high *CCR7* and low let-7a expression levels, whereas KYSE-590 had significantly low *CCR7* and high let-7a expression levels. The expression levels of the *CCR7* and let-7a were normalized based on the expression levels in KYSE-350 cell, which were defined as 1 (Table S1).

### Relationship between let-7a and *CCR7* expression in ESCC cells

**Fig. 2:**
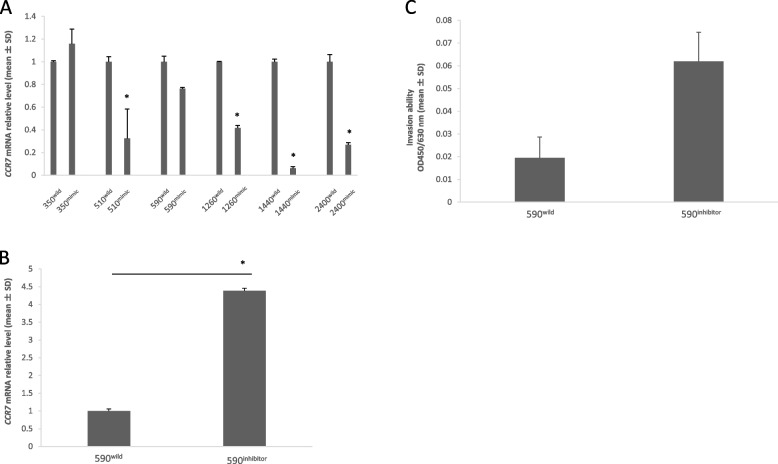
Changes in *CCR7* expression levels and the invasive ability of KYSE cells due to the regulation of let-7a expression. **A** Overexpression of let-7a decreased *CCR7* expression levels in five out of six KYSE cell lines. (* *p* < 0.05 versus wild-type). **B** Downregulation of let-7a increased *CCR7* expression levels in KYSE 590 cells (*p* < 0.001). (* *p* < 0.05 versus wild-type). C) Increase in invasion ability of KYSE 590 cells after downregulation of let-7a (*p* = 0.061)

As determined via quantitative RT-PCR analysis, the expression of *CCR7* was downregulated by the transfection of let-7a in five of the six KYSE cell lines (Fig. 2A). The decrease in *CCR7* expression level was most prominent in KYSE-1440; *CCR7* expression level was the highest in the wild-type.

### Downregulation of let-7a increases *CCR7* expression level and promotes invasion ability in KYSE-590 cells

The inhibitory effect of let-7a was determined by using synthetic anti-let-7a oligonucleotides in KYSE-590 esophageal cancer cells, which expressed a low level of *CCR7* and a high level of let-7a in the wild-type. After transfection with synthetic anti-let-7a oligonucleotides, the level of *CCR7* expression in KYSE-590 cells increased (*p* < 0.001; Fig. 2B), along with their invasive ability (*p* = 0.061; Fig. 2C).

### Development of highly invasive cell lines and lymph node metastasis model

**Fig. 3:**
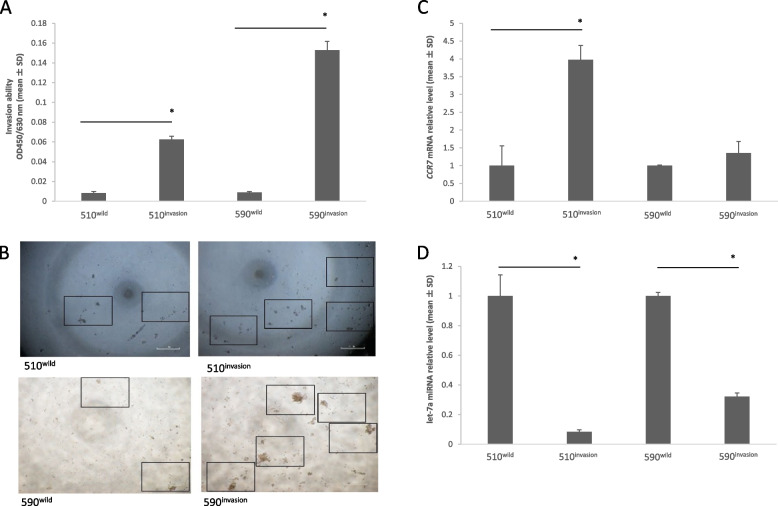
Invasive ability of and *CCR7* and let-7a expression in KYSE-510 cells after six invasion assays. **A** Increased invasive ability after invasion assay (KYSE-510: *p* < 0.001, KYSE-590: *p* = 0.001). **B** Increased number of KYSE-510 and -590 cells migrated after invasion assay (cells are shown in the area surrounded by square). **C** Increased expression level of *CCR7* in synthesized highly invasive cell lines (KYSE-510: *p* = 0.004, KYSE-590: *p* = 0.203). **D** Decreased expression level of let-7a in synthesized highly invasive cell lines (KYSE-510: *p* < 0.001, KYSE-590: *p* = 0.001). (* *p* < 0.05 versus wild-type)

After six courses of the invasion assay, the invasive ability of the highly invasive lines increased (KYSE-510: *p* = 0.004, KYSE-590: *p* = 0.001, Fig. 3A–B). Compared to that in the wild-type, *CCR7* expression level increased (KYSE-510: *p* = 0.04, KYSE-590: *p* = 0.203, Fig. 3C) in the invasive type, but let-7a expression level decreased (KYSE-510: *p* < 0.001, KYSE-590: *p* = 0.001, Fig. 3D).

**Fig. 4:**
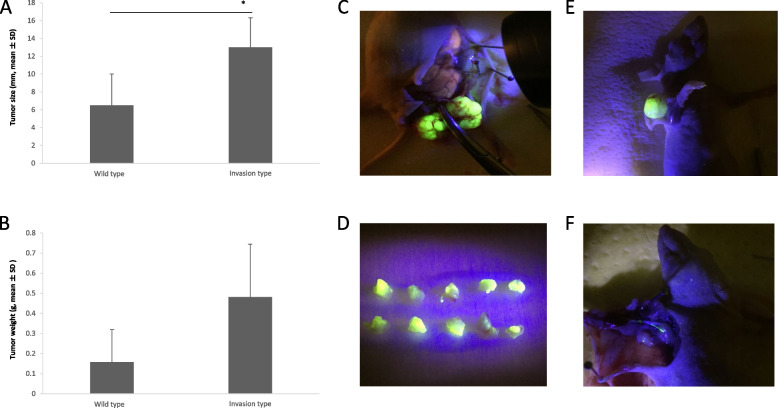
Development of lymph node metastasis model. **A** Tumor size corresponding to invasive and wild-type KYSE-510 cells (*p* = 0.025). **B** Tumor mass corresponding to invasive and wild-type KYSE-510 cells (*p* = 0.070). **C** Subcutaneous tumor established via xenograft injection. **D** Xenograft extracted and excised into 2 × 2 mm pieces, visualized with GFP. **E** Subdivided xenograft transplanted and grown in mouse elbow, visualized with GFP. **F** Axillary lymph node metastasis via lymphatic vessels. (* *p* < 0.05 versus wild-type)

Since the KYSE-510 cell line alone could be injected into mice for the in vivo experiment, we used it for the mouse model. The implanted primary tumor of invasive KYSE-510 was both larger (6.5 ± 3.51 vs. 13.0 ± 3.31 mm, *p* = 0.025) and heavier (0.158 ± 0.161 vs. 0.481 ± 0.263 g, *p* = 0.070) than that of wild-type cells (Fig. 4A–D). Similarly, the percentage of positive lymph node metastasis of the invasive-type cells, labeled with GFP, was higher than that of the wild-type — 25% (1/4) and 0% (0/5), respectively (Fig. 4E–F).

### Relationship between *CCR7* and let-7a expression in ESCC patients

**Fig. 5:**
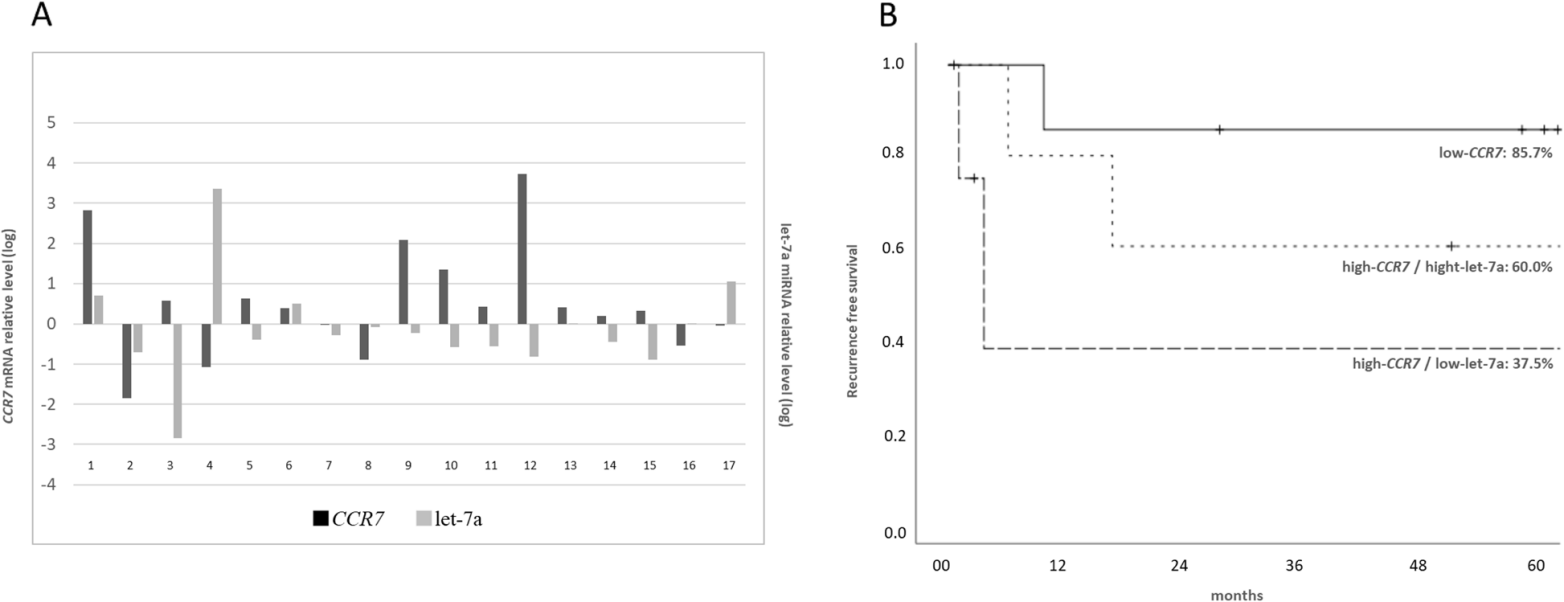
Relationship between *CCR7* and let-7a expression and the prognostic implication. **A** *CCR7* and let-7a expression levels in ESCC patients. **B** Recurrence-free survival curve for ESCC patients in the low-*CCR7*, high-*CCR7*/high-let-7a, and high-*CCR7*/low-let-7a groups (*p* = 0.038)

Tissue samples were obtained from 17 ESCC patients during a biopsy, and their characteristics are listed in Table 1. Figure 5A shows the expression levels of *CCR7* and let-7a in cancer tissue relative to those in normal tissue, defined as 1 based on quantitative RT-PCR. The graph uses a logarithmic notation, thus presenting negative values when the *CCR7* and let-7a expression level ratio is less than 1. In 11 ESCC patients, *CCR7* expression levels were higher in malignant tissues than in normal tissues (positive value). In contrast, in a different group of 11 ESCC patients, let-7a expression was downregulated more in malignant tissues than in normal tissues (negative value). Around nine ESCC patients exhibited a negative correlation between *CCR7* and let-7a expression.

**Table 1:** Background characteristics and pathological features of patients with esophageal cancer

Variables	esophageal cancer patients *n* = 17
Gender	
Male	14
Female	3
Age (median, range)	69 (35–75)
UICC TNM 8th	
Pathological T factor	
T1	3
T2	1
T3	13
Pathological N factor	
N0	6
N1	4
N2	7
Pathological Stage	
Stage I	1
Stage II	7
Stage III	9

*UICC* Union for international cancer control tnm classification of malignant tumors (8th edition)

### The impact of *CCR7* and let-7a expression on prognosis

Recurrence of tumor was observed in six patients, and the most predominant sites were the lymph nodes. Using the median value, patients were split into two respective groups with low and high *CCR7* expression levels; patients with high *CCR7* expression levels were further divided into two sub-groups with low and high let-7a expression levels. The high-*CCR7*/low-let-7a group (5-RFS: 37.5%) had the worst prognosis, followed by the high-*CCR7*/high-let-7a (5-RFS: 60.0%), and then the low-*CCR7* group (5-RFS: 85.7%; *p* = 0.038, Fig. 5B). Cox proportional hazards analysis identified the high-*CCR7*/low-let-7a group as a significant prognostic factor (Table 2).

**Table 2:** Univariate Cox proportional hazard analysis

	Univariate		
Variables	HR	95% CI	*p*-value
Age			0.547
< 70	1.000		
≥ 70	1.644	0.326–8.306	
Pathological T factor			0.381
T1-2	1.000		
T3-4	2.639	0.302–23.083	
Pathological N factor			0.831
negative	1.000		
positive	0.831	0.152–4.552	
*CCR7* high / Let-7a low			0.028
None	1.000		
Yes	6.476	1.222–34.309	

*HR* Hazard ratio, *CI* Confidence intervals

## Discussion

This study showed that synthetic let-7a and the let-7a inhibitor respectively decreased and increased *CCR7* expression levels in KYSE cells, demonstrating a negative correlation between *CCR7* and let-7a expression. Therefore, the downregulation of let-7a miRNA is a factor contributing to increased *CCR7* expression levels in esophageal cancer cells and the development of malignant tumors.

High *CCR7* expression level is related to lymphatic tumor metastasis and prognosis in gastric as well as colorectal cancer [16, 17]. Crucially, the elevated *CCR7* expression level caused by a deficiency in let-7a expression level is intricately linked to the metastasis and progression of cancer [12]. Furthermore, *CCR7* is correlated with lymphatic metastasis and poor prognosis in different types of breast cancer, while let-7a has been found to suppress breast cancer cell migration and invasion through the downregulation of *CCR7* expression [10, 18]. Thus, our study confirmed the negative correlation between *CCR7* and let-7a expression in ESCC, which strongly suggests that let-7a, suppresses the expression of *CCR7*. Regarding the direct interaction between *CCR7* and let-7a, Kim et al. used the luciferase assay to show that let-7a directly regulates *CCR7* expression by binding with its 3′-UTR [10]. In addition, we can check what type of gene sequence can be regulated by specific miRNA at Target Scan Human 8.0 [19]; it showed that *CCR7* is regulated by let-7a binding its 3′-UTR.

In normal human cells, *CCR7* is mainly expressed in differentiated lymphocytes as well as the surface of dendritic cells, and it is thought to mediate lymphocyte migration [20]. The invasive ability of cancer cells is affected by the expression of *CCR7* [10, 18]. In this study, the invasive ability of ESCC increased after transfection with synthetic anti-let-7a because of the increased and decreased *CCR7* and let-7a expression levels, respectively. After the invasion assay, the expression level of *CCR7* in the invasive-type cells increased while that of let-7a decreased, in comparison with those in the wild-type. Therefore, the respective increase and decrease in *CCR7* and let-7a expression levels are important factors affecting the invasive ability of ESCC. We also used the highly invasive cell line for in vivo experiments on mice. The implanted primary tumor of the invasive-type was larger in size and mass than that of the wild-type, and the percentage of positive lymph node metastasis was higher as well. Therefore, the highly invasive cell line was more likely to cause and accelerate lymph node metastasis in vivo.

We also investigated the expression of *CCR7* and let-7a miRNA in ESCC patient tissues and demonstrated that they tend to have a higher *CCR7* and a lower let-7a miRNA expression levels than normal tissues. Furthermore, we found that the expression levels of *CCR7* and let-7a miRNA can stratify the prognosis of the ESCC patients. Patients in the two high-*CCR7* groups had a worse prognosis than those in the low-*CCR7* group, with the low-let-7a group exhibiting a worse prognosis than the high-let-7a group. These results suggest that during carcinogenesis, the suppression of *CCR7* expression diminishes owing to the decreased expression level of let-7a, and that these changes may affect the prognosis of ESCC patients. Supporting this finding, He et al. [15] found that the plasma levels of let-7a miRNA were significantly lower in ESCC patients than in healthy participants.

However, our study has three main limitations. First, while we confirmed the negative correlation between *CCR7* and let-7a, we did not account for the potential influence of other oncogenes controlled by let-7a. Second, in the mouse experiment, we demonstrated the in vivo malignancy of the synthesized highly invasive cell line but did not measure *CCR7* or let-7a expression levels in metastatic lymph nodes. Nevertheless, the relationship between *CCR7* expression level and lymph node metastasis in ESCC cells has been examined in a previous study [6]. Third, the number of cell lines and patient tissue samples used in this study was relatively small; therefore, additional studies are required to validate these results.

## Conclusions

The expression of *CCR7* was found to be downregulated by let-7a miRNA in ESCC cells. Thus, a decrease in let-7a expression level led to an increase in the expression level of *CCR7* in ESCC cells, which consequently acquired increased invasive ability and malignancy, resulting in a worse prognosis for patients with ESCC.

## Supplementary Information

**Additional file 1:** **Table S1.** Quantitative RT-PCR analysis.

## Data Availability

The datasets KYSE-350, 510, 590, 1260, 1440, and 2400 for this study can be found and are available in the JCRB cell bank (https://cellbank.nibiohn.go.jp/english). https://cellbank.nibiohn.go.jp/~cellbank/en/search_res_det.cgi?RNO=JCRB1428 https://cellbank.nibiohn.go.jp/~cellbank/cgi-bin/search_res_det.cgi?ID=6561 https://cellbank.nibiohn.go.jp/~cellbank/cgi-bin/search_res_det.cgi?ID=6572 https://cellbank.nibiohn.go.jp/~cellbank/en/search_res_det.cgi?RNO=JCRB1434 https://cellbank.nibiohn.go.jp/~cellbank/cgi-bin/search_res_det.cgi?ID=6557 https://cellbank.nibiohn.go.jp/~cellbank/cgi-bin/search_res_det.cgi?ID=6542 The datasets of miRNAs are available in the miRbase (https://www.mirbase.org/). https://www.mirbase.org/cgi-bin/mature.pl?mature_acc=MIMAT0000062 https://www.mirbase.org/cgi-bin/mature.pl?mature_acc=MIMAT0000039

## Abbreviations

3′-UTR: 3′-Untranslated region
CCR7: C–C chemokine receptor type 7
cDNA: Complementary DNA
dsRNA: Double-stranded RNA
ESCC: Esophageal squamous cell carcinoma
GAPDH: Glyceraldehyde-3-phosphate dehydrogenase
GFP: Green fluorescent protein
miRNA: MicroRNA
RFS: Recurrence-free survival
RT-PCR: Reverse-transcription polymerase chain reaction
